# Fatigue and associated factors in myasthenia gravis: a nationwide registry study

**DOI:** 10.1007/s00415-024-12490-2

**Published:** 2024-06-13

**Authors:** Mattea Funke, Maria Eveslage, Jana Zschüntzsch, Tim Hagenacker, Tobias Ruck, Charlotte Schubert, Michael Schroeter, Andreas Meisel, Heinz Wiendl, Sarah Hoffmann, Jan D. Lünemann

**Affiliations:** 1 Department of Neurology with Institute of Translational Neurology, University Hospital Münster, 48149 Münster, Germany; 2 Institute of Biostatistics and Clinical Research, University of Münster, 48149 Münster, Germany; 3 Department of Neurology, University Medical Center Göttingen, Georg-August University, 37075 Göttingen, Germany; 4 Department of Neurology, Center for Translational Neuro- and Behavioral Sciences (C-TNBS), University Hospital Essen, Hufelandstr. 55, 45147 Essen, Germany; 5 Department of Neurology, Medical Faculty and University Hospital Düsseldorf, Heinrich Heine University Düsseldorf, Düsseldorf, Germany; 6 Institute of Neuroimmunology and Multiple Sclerosis (INIMS), University Medical Center Hamburg-Eppendorf, Hamburg, Germany; 7 Department of Neurology, University Cologne and University Hospital, Cologne, Germany; 8 Charité – Universitätsmedizin, Corporate Member of Freie Universität Berlin and Humboldt-Universität zu Berlin, Center for Stroke Research Berlin, Charitéplatz 1, 10117 Berlin, Germany; 9 Berlin Institute of Health at Charité – Universitätsmedizin Berlin, Charitéplatz 1, 10117 Berlin, Germany

## Abstract

Fatigue is commonly associated with myasthenia gravis (MG), but factors contributing to fatigue development in MG are incompletely understood. This nationwide cross-sectional registry study included 1464 patients diagnosed with autoimmune MG, recruited between February 2019 and April 2023. Frequency and severity of fatigue was assessed at study inclusion using the patient-reported Chalder Fatigue Questionnaire (CFQ). Frequency of fatigue was 59%. Fatigue severity strongly correlated with both patient-reported and physician-assessed MG outcome measures (MG-ADL, MG-QoL15, QMG and MGFA classes) and was associated with a history of myasthenic exacerbation and/or myasthenic crises and a delay in diagnosis of more than 1 year after symptom onset. Fatigue was more prevalent in women and coincided with symptoms of depression, anxiety, and sleep dissatisfaction. Differences in fatigue severity were observed between antibody (ab) subgroups, with highest fatigue severity in LRP4-ab-positive patients and lowest fatigue severity in AChR-ab-positive patients. Fatigue is a frequent and clinically highly relevant symptom of MG. Early diagnosis and prevention of MG crises may limit the long-term burden of fatigue in patients with MG.

**Supplementary Information:**

The online version contains supplementary material available at 10.1007/s00415-024-12490-2.

Dear Sirs,

Myasthenia gravis (MG) is an autoimmune disease in which immunoglobulin G autoantibodies (Abs) bind to acetylcholine receptors (AChR) or to functionally related molecules at the postsynaptic membrane of the neuromuscular junction, leading to localized or general fatigable muscle weakness [1]. In recent years, several studies have shown that MG patients are also affected by excessive mental and physical exhaustion, that differs from muscle fatigability, commonly referred to as fatigue [2, 3]. While fatigue is a symptom in many chronic diseases, previous studies reported particularly high prevalence rates, up to 80%, in MG patients [2–5]. Here, we studied a large real-world representative German registry cohort to assess fatigue and fatigue-associated factors in patients with MG.

The German National MG registry (MyaReg) is a multicenter prospective observational study comprising detailed assessment of patients diagnosed with a myasthenic syndrome using a standardized protocol across 19 certified German MG Centers, accredited by the German Myasthenia Society (Deutsche Myasthenie Gesellschaft, DMG). Certified centers fulfill patient-centered quality requirements and form a network for the continuous improvement of management and treatment standards in MG. In this study, only patients with confirmed diagnosis of autoimmune MG (excluding patients with Lambert–Eaton Myasthenic Syndrome or Congenital Myasthenic Syndrome) were included (*n* = 1464). Diagnostic criteria were adhering to the current German guidelines: The diagnosis of MG is based on the history and physical findings of fatigable and fluctuating muscle weakness. The diagnosis is confirmed by positive findings in auto-Ab diagnostics and/or electrophysiology and/or pharmacological testing [6]. MyaReg is registered in the German clinical trial registry (https://www.drks.de, DRKS00024099, first patient enrolled: February 4th, 2019). This cross-sectional study was approved by the ethics committee of the Charité-Universitätsmedizin Berlin, Germany (EA1/214/18), and subsequently by all local ethics committees of the participating centers. It was conducted in accordance with the STROBE guidelines, with the checklist provided in Suppl. 3. All patients provided written informed consent. Data were retrieved on April 12, 2023. Detailed information on physician-assessed and patient-reported MG-specific outcome measures used in this study are provided in Suppl. 1.

Fatigue severity was measured using the Chalder Fatigue Questionnaire (CFQ), a self-assessment tool comprising 11 items addressing both physical and mental fatigue. Respondents rated their experiences on a 4-point scale (“less than usual” to “much more than usual”). Two scoring systems, Likert scoring (0–1-2–3, total score 0–33) and bimodal scoring (0–0-1–1, total score 0–11), are commonly employed, with the latter allowing for categorization of fatigue caseness based on a cut-off score of 4 points or more [7]. Bimodal scoring was used to calculate fatigue prevalence in the study population and Likert scoring for all subsequent investigations on factors associated with fatigue.

Data are presented as *n* (%), with mean (Standard Deviation, SD) for normally distributed numbers and median (mdn) (Interquartile Range, lower–upper quartile, IQR) for skewed distributions. Spearman Rank’s Correlation Coefficient (*r*) was utilized for correlation analyses involving non-normally distributed data, specifically between fatigue score and MG-related standardized scores. Multiple linear regression analysis was conducted to analyze and weigh potential factors influencing fatigue, which was the primary outcome of interest. Depression, anxiety, and insomnia were considered potential confounders, their associations with disease severity and fatigue are highlighted in the discussion section. In univariable analyses, non-parametric Mann–Whitney *U* tests were employed, exploring differences in fatigue severity concerning the exposure variables sex, symptoms, history of MG exacerbation/crisis, and time to diagnosis. In multivariable analyses, particularly when examining differences between antibody subgroups or MGFA classification, Kruskal–Wallis tests were applied. All analyses were performed without imputation methods (i.e. complete case analysis), using original data to ensure transparency and maintain integrity of the results. *p* values ≤ 0.05 are considered significant. As no adjustment for multiple testing was applied, all analyses must be interpreted as exploratory. Statistical analyses were carried out using IBM SPSS Statistics 29. Figures were generated using R version 4.2.2, and assembled using Inkscape 1.2.2 (dated 2022–12-05).

Baseline characteristics of the 1464 participants (830 women [56.7%]; mean [SD] age, 58.4 [17] years) are displayed in Table 1. In total, 58.8% (*n* = 611) of the patients experienced symptoms of fatigue as defined by the CFQ. Fatigue strongly correlated with disease severity as measured by all patient-assessed and physician-reported outcome measures (MG-ADL, MG-QoL15, QMG as well as MGFA classification) (Fig. 1A).

**Table 1:** Baseline demographics and clinical characteristics

Characteristic	No. (%)	Available sample size *n* (total = 1464)
Age, mean, (SD), years	58 (17)	1463
Sex		1464
Female	830 (56.7)	
Male	629 (43)	
Non-binary	5 (0.3)	
Antibody status		
AChR+	755 (67.5)	1118
MuSK+	31 (2.8)	1118
LRP4+	10 (1.5)	679
Triple-seronegative	284 (25.4)	1118
AChR+MuSK+	24 (2.1)	1118
AChR+LRP4+	14 (2.1)	679
Symptoms		
Ocular	1178 (82.7)	1424
Bulbar	674 (47.8)	1411
Myalgia	345 (24.3)	1418
History of myasthenic crisis/exacerbation	169 (37.6)	450
Outcome measures, median, (IQR)		
CFQ	16 (11–21)	1464
CFQ ≥ 4, No., (%)	610 (58.8)	1037
QMG	5 (2–10)	628
MG-QoL15	14 (4.25–26.75)	1308
MG-ADL	4 (1–7)	1253
HADS total	11.5 (7.8)	1381
HADS-A	6 (3–9)	990
HADS-A ≥ 8, No., (%)	353 (35.7)	
HADS-D	4 (2–8)	991
HADS-D ≥ 8, No. (%)	276 (27.9)	
ISI	8 (3.75–14)	1390
ISI ≥ 10, No., (%)	622 (44.7)	
Current MGFA class, No. (%)		1090
I ocular weakness	272 (25)	
II mild generalized weakness	650 (59.6)	
III moderate generalized weakness	150 (13.8)	
IV severe generalized weakness	16 (1.5)	
V intubation	2 (0.2)	
Post-intervention Status		258
Change in status improved	65 (25)	
Complete stable remission	3 (1.2)	
Exacerbation	1 (0.4)	
Minimal Manifestations MM-0	5 (1.9)	
MM-1	8 (3.1)	
MM-2	11 (4.3)	
MM-3	50 (19.4)	
Pharmacologic remission	5 (1.9)	
Unchanged	105 (40.7)	
Worse	5 (1.9)	
Duration of disease, median, (IQR), years	5 (2–11)	1318
Time to diagnosis, median, (IQR), months	3 (1–14)	1304
Medication		
Acetylcholinesterase inhibitors	1413 (96.7)	1461
Glucocorticoids	1103 (75.9)	1454
Non-steroidal immunosuppressants	990 (67.8)	1461

*AChR* acetylcholine receptor, *current MGFA class* Myasthenia Gravis Foundation of America classification at examination date, *CFQ* Chalder Fatigue Questionnaire, *HADS* Hospital Anxiety and Depression Scale, *ISI* Insomnia Severity Index, *IQR* interquartile range, *LRP4* lipoprotein receptor-related protein 4, *MG-ADL* Myasthenia Gravis Activities of Daily Living scale, *MG-QoL15* Myasthenia Gravis Quality-of-Life15 score, *MuSK* muscle-specific tyrosine kinase, *QMG* Quantitative Myasthenia Gravis score, *SD* standard deviation

**Fig. 1:**
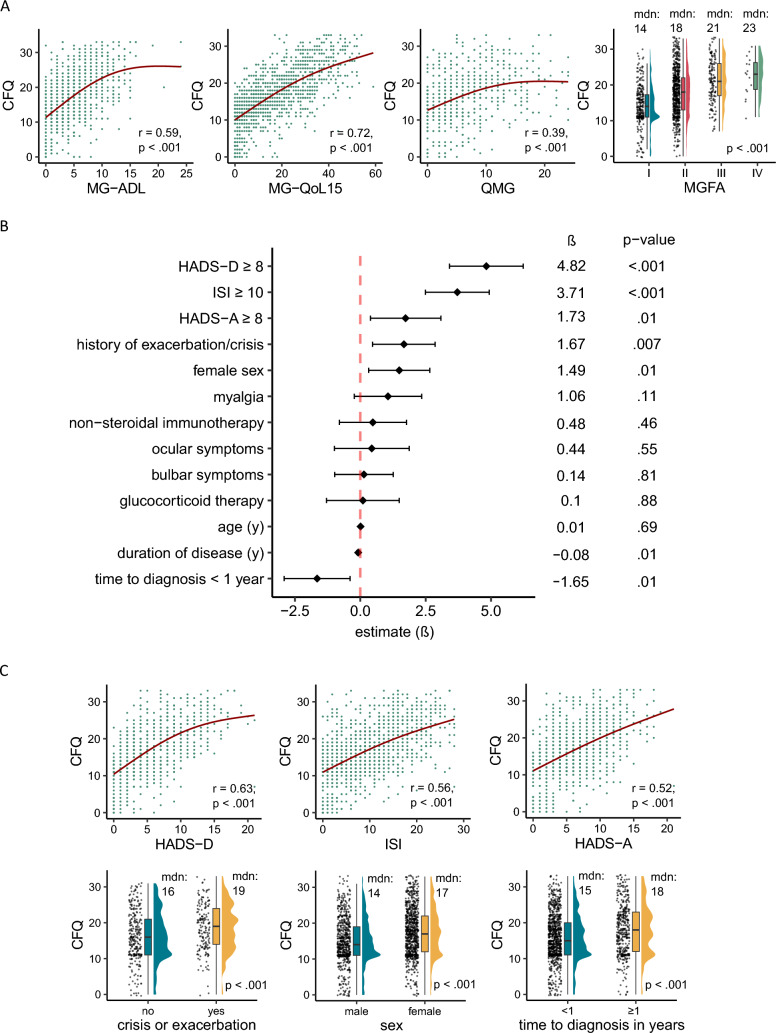
Association of MG-related outcome measures, demographical and clinical features with fatigue severity. **A** Spearman’s Rank Correlation analysis of MG-ADL, MG-QoL15 and QMG with fatigue score, displayed as scatterplots with trendlines, and Kruskal–Wallis test comparing MGFA classes with respect to their median (mdn) fatigue scores. **B** Results of multiple linear regression analysis (*n* = 1464) with fatigue severity (CFQ) as dependent variable and demographical and disease-related features as independent variables; *F*-Ratio = 20.5, *p* < .001, *R*^2^ = 0.43. The unstandardized regression coefficient (*β*) with 95% confidence interval indicates how much the dependent variable changes when the predictor variable changes by one unit. **C** Spearman’s Rank Correlation analysis of HADS-Depression (HADS-D), sleep disorders (ISI) and HADS-Anxiety (HADS-A) with fatigue severity, presented as scatterplots with trendlines, and univariate analyses comparing median fatigue scores in relation to sex, history of crises/exacerbations and time to diagnosis < / > 1 year. MGFA, current Myasthenia Gravis Foundation of America classification at examination date; CFQ, Chalder Fatigue Questionnaire; HADS, Hospital Anxiety and Depression Scale; ISI, Insomnia Severity Index; MG-ADL, Myasthenia Gravis Activities of Daily Living scale; MG-QoL15, Myasthenia Gravis Quality-of-Life15 score; QMG, Quantitative Myasthenia Gravis score

In multiple regression analysis as well as subsequent univariable analyses, female sex, presence of symptoms of depression and anxiety as assessed by HADS-D/HADS-A-score ≥ 8 points, sleep disturbances as measured by the Insomnia Severity Index (ISI) ≥ 10 points, history of MG exacerbation/crisis, and time to diagnosis of more than 1 year after symptom onset were significantly associated with higher fatigue severity (Fig. 1B, C). Age, ocular, bulbar symptoms, myalgia, and immunotherapy with corticosteroids or non‐steroidal immunosuppressants were not significantly associated with fatigue severity (Fig. 1B).

Fatigue severity varied significantly among MG antibody (ab) subtype groups. LRP4-ab-positive patients demonstrated the highest fatigue scores (mdn CFQ 20.5, SD 9.2), whereas AChR-ab-positive patients exhibited the lowest fatigue scores (mdn CFQ 15, SD 6.7). MuSK-ab-positive patients had median CFQ scores of 16 (SD 6.4), whereas seronegative patients had median CFQ scores of 18 (SD 6.7) (Suppl. 2).

Our study demonstrates that a history of MG exacerbation/crisis and a delayed diagnosis of MG are associated with higher fatigue severity in MG, apart from previously reported associated factors, such as female sex, depressive symptoms, and sleep dissatisfaction [2–5, 8–10]. Fatigue is common in MG and strongly correlates with disease severity and quality of life. The presence of specific antibodies influences fatigue severity, with highest fatigue levels in LRP4-ab-positive patients and lowest fatigue levels in AChR-ab-positive patients. However, the number of cases in the LRP4-ab-positive group was very low (*n* = 10), so this data has to be interpreted with caution. Large case numbers were available for AChR-ab-positive (*n* = 755) and seronegative (*n* = 284) MG patients, showing higher CFQ scores in seronegative patients compared to AChR-ab-positive patients.

A symptom cluster of fatigue, depression, anxiety, and sleep disturbances appears not to be specific to MG as it is also observed in other chronic diseases, including cancer [11–13], highlighting the complex interrelations and the need for exploring underlying biological mechanisms and effective therapies for fatigue. Chronic low-grade activation of inflammatory pathways, as seen in autoimmune diseases such as MG, has been suggested to contribute to persistent fatigue and fatigue-associated symptoms [14]. Depression, anxiety, and sleep disturbances are also related to disease severity, making it challenging to determine causal relationships between these factors and fatigue severity.

Two previously unrecognized modifiable factors were specifically related to fatigue severity in MG: a history of MG exacerbation/crisis and a delay in diagnosing MG. Despite therapeutic advances, up to 20% of patients with MG experience a myasthenic crisis during their disease course, a life-threatening complication characterized by worsening of muscle weakness, potentially resulting in respiratory failure requiring intubation and mechanical ventilation. Psychological sequelae of experiencing a MG crisis are poorly understood. Supporting the concept that a history of MG crisis may trigger long-term perception of fatigue and related symptoms, patients followed up for 1 year after experiencing MG-related respiratory insufficiency reportedly suffer from increased burden of fatigue, symptoms of anxiety, depression, and fear of a new crisis [15]. Early and effective management of MG exacerbations, based on increased awareness of these risk factors may not only reduce crisis-associated mortality rates but also prevent the development of fatigue in the long term.

MG is a rare disease and its phenotype can be remarkably heterogeneous. Awareness of symptoms and signs of MG among non-specialists is often limited, frequently resulting in delays in diagnosing MG [16]. Due to the absence of specific antibodies, seronegative patients often receive their diagnosis and start of treatment considerably later than AChR-ab-positive patients, exposing them at heightened risk for increased fatigue severity. Disease activity in MG is most prominent within the first few years after onset, while access and response to immunotherapy within the first year are associated with improved long-term outcome. Early diagnosis within 1 year after symptom onset was associated with lower fatigue severity in our study, highlighting the long-term clinical impact of achieving an early diagnosis, potentially early access to effective immunotherapy and in limiting MG-related disease burden.

Limitations of our study include the cross-sectional design as it does not provide information on causality and the effect of therapy-induced clinical disease remission on fatigue and fatigue-associated symptoms. BMI and physical exercise have been known to influence fatigue and overall health outcomes in MG patients. The absence of this data, along with information on refractory MG cases, represents a limitation in our study. We recommend including BMI, physical exercise habits, and refractory MG in future studies to enhance our understanding of MG. The strong correlation with disease severity suggests that clinically meaningful sustained improvement, reportedly achievable by newly approved medications, such as complement inhibitors and neonatal Fc receptor agonists, will substantially affect fatigue severity. Prospective longitudinal studies are required to better identify risk factors for developing fatigue and effective strategies to limit fatigue-associated disease burden in MG.

### Supplementary Information

Below is the link to the electronic supplementary material.

Supplementary file1 (DOCX 152 KB)

## Data Availability

Data will be made available by the corresponding author upon reasonable request.
