# Supplementary material for: Fatigue and associated factors in myasthenia gravis: a nationwide registry study
Source: J Neurol. 2024 Jun 13;271(8):5665–70. doi: 10.1007/s00415-024-12490-2 (PMC11319509; doi:10.1007/s00415-024-12490-2)
Supplement: Supplementary file 1 — Supplementary file1 (DOCX 152 KB) [file 415_2024_12490_MOESM1_ESM.docx]

**Supplemental Material:**

**Suppl. 1 MG outcome measures**

Physician-assessed outcome measures:

*Myasthenia Gravis Foundation of America (MGFA) classification:* The MGFA classification divides MG into five classes, based on clinical features and disease severity, primarily related to the affected muscles. It distinguishes between ocular MG (MGFA I) and generalised MG (MGFA II-V), with generalised MG further divided into mild (MGFA II), moderate (MGFA III) and severe (MGFA IV) generalised MG. MGFA V describes a state of myasthenic crisis requiring intubation^1^.

*Quantitative Myasthenia Gravis (QMG) score:* The QMG score is a clinical tool for assessing the severity of MG in patients. It consists of 13 items evaluating clinical features of MG including muscle strength, ocular symptoms, bulbar and respiratory function. The total QMG score ranges from 0 to 39 and shows good interrater reliability^1,2^.

Patient-reported outcome measures:

*MG-quality of life 15 (MG-QoL15) scale:* The MG-QoL15 scale is a self-reported questionnaire consisting of 15 questions covering different aspects of health-related quality of life such as physical and social functioning, emotional well-being and treatment burden. Each question is scored on a 5-point scale, ranging from 0 (“not at all”) to 4 (“very much”) with a total sum score of 60^3^.

*MG-activities of daily living (MG-ADL) scale:* The MG-ADL scale assesses the impact of MG on functions like talking, chewing, swallowing, breathing, brushing teeth or hair, arising from chair and double vision eyelid droop. Each item is scored on a 4-point scale from 0 (normal) to 3 (most severe), with a total score range of 0-24^4^.

*Chalder Fatigue Questionnaire (CFQ):* The CFQ is a self-assessment tool consisting of 11 items, including 7 items addressing physical fatigue and 4 items focusing on mental fatigue. Respondents rate their experiences on a 4-point scale, ranging from “less than usual”, no more than usual”, “more than usual” to “much more than usual”, leading to a total sum score of 33 points. To analyse the questionnaire responses, two scoring systems are commonly employed: bimodal scoring (0-0-1-1, total score 0-11) and likert scoring (0-1-2-3, total score 0-33). To define fatigue caseness, a cut-off value of 4 items or more is recommended in bimodal scoring system^5^.*Top of FormBottom of Form*

*Hospital Anxiety and Depression Scale (HADS):* HADS is a questionnaire consisting of 14 items, with 7 items assessing symptoms of anxiety (HADS-A) and 7 items examining symptoms of depression (HADS-D). The maximum score is 24 for each subscale and 42 in total. A cut-off score of 8 and more for each subscale is used to define cases of anxiety or depression^6^.

*Insomnia Severity Index (ISI):* ISI is used to assess the severity of sleep disorders over the previous two weeks. It consists of 7 items, including difficulty falling asleep, difficulty staying asleep, or waking up too early and feeling tired during the day. The maximum total score is 28 and a cut-off score of 10 or more is used to define caseness^7,8^.

Abbreviations:

AChR, acetylcholine receptor; MGFA, Myasthenia Gravis Foundation of America classification; CFQ, Chalder Fatigue Questionnaire; HADS, Hospital Anxiety and Depression Scale; ISI, Insomnia Severity Index; LRP4, lipoprotein receptor-related protein 4; MG-ADL, Myasthenia Gravis Activities of Daily Living score; MG-QoL15, Myasthenia Gravis Quality-of-Life15 score; MuSK, muscle-specific tyrosine kinase; QMG, Quantitative Myasthenia Gravis score.

**Suppl. 2 MG antibody profile and fatigue severity**


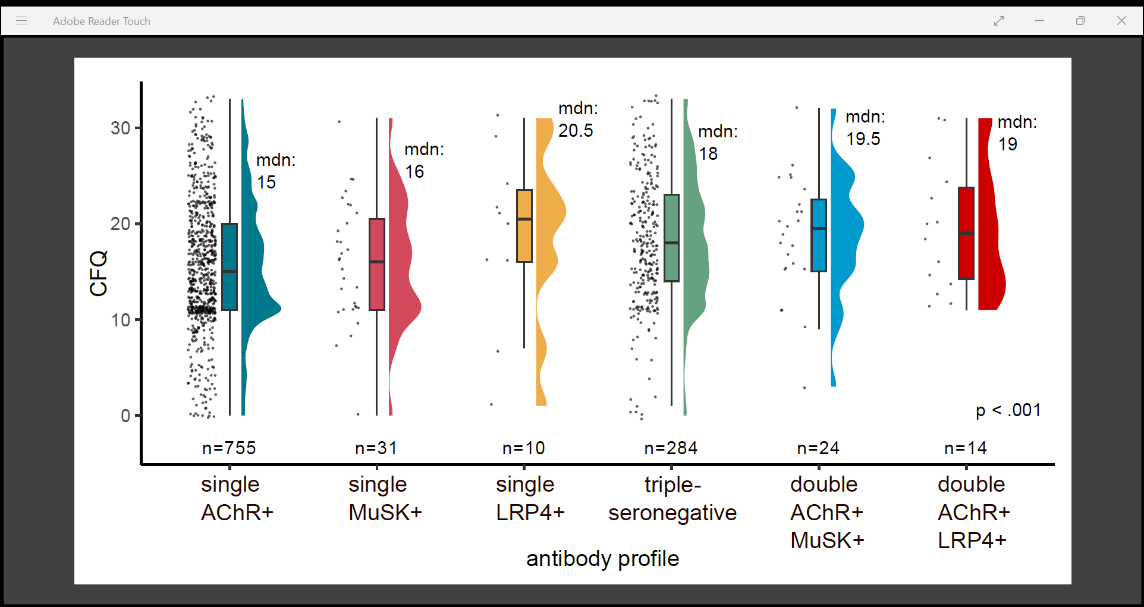


Abbreviations:

AChR+, acetylcholine receptor antibody positive; CFQ, Chalder Fatigue Questionnaire; LRP4+, lipoprotein receptor-related protein 4 antibody positive; mdn, median; MuSK+, muscle-specific tyrosine kinase antibody positive; triple-seronegative, AChR-, MuSK-, LRP4-antibody-negative.

**Suppl. 3 STROBE Statement — Checklist of items that should be included in reports of *cross-sectional studies***

|  | Item No | Recommendation |  | Page No |  |
| --- | --- | --- | --- | --- | --- |
| **Title and abstract** | 1 | (*a*) Indicate the study’s design with a commonly used term in the title or the abstract |  | 1, 2 |  |
|  |  | (*b*) Provide in the abstract an informative and balanced summary of what was done and what was found |  |  |  |
| Introduction | | |  |  |  |
| Background/rationale | 2 | Explain the scientific background and rationale for the investigation being reported |  | 3 |  |
| Objectives | 3 | State specific objectives, including any prespecified hypotheses |  | 3 |  |
| Methods | | |  |  |  |
| Study design | 4 | Present key elements of study design early in the paper |  | 4 |  |
| Setting | 5 | Describe the setting, locations, and relevant dates, including periods of recruitment, exposure, follow-up, and data collection |  | 4 |  |
| Participants | 6 | (*a*) Give the eligibility criteria, and the sources and methods of selection of participants |  | 4 |  |
| Variables | 7 | Clearly define all outcomes, exposures, predictors, potential confounders, and effect modifiers. Give diagnostic criteria, if applicable |  | 4, 5 |  |
| Data sources/ measurement | 8* | For each variable of interest, give sources of data and details of methods of assessment (measurement). Describe comparability of assessment methods if there is more than one group |  | 4 |  |
| Bias | 9 | Describe any efforts to address potential sources of bias |  |  |  |
| Study size | 10 | Explain how the study size was arrived at |  | n.a. |  |
| Quantitative variables | 11 | Explain how quantitative variables were handled in the analyses. If applicable, describe which groupings were chosen and why |  | 5 |  |
| Statistical methods | 12 | (*a*) Describe all statistical methods, including those used to control for confounding |  | 5 |  |
|  |  | (*b*) Describe any methods used to examine subgroups and interactions |  |  |  |
|  |  | (*c*) Explain how missing data were addressed |  |  |  |
|  |  | (*d*) If applicable, describe analytical methods taking account of sampling strategy |  |  |  |
|  |  | (*e*) Describe any sensitivity analyses |  |  |  |
| Results | | |  |  |  |
| Participants | 13* | (a) Report numbers of individuals at each stage of study—eg numbers potentially eligible, examined for eligibility, confirmed eligible, included in the study, completing follow-up, and analysed |  | 6 |  |
|  |  | (b) Give reasons for non-participation at each stage |  |  |  |
|  |  | (c) Consider use of a flow diagram |  |  |  |
| Descriptive data | 14* | (a) Give characteristics of study participants (eg demographic, clinical, social) and information on exposures and potential confounders |  | 6 |  |
|  |  | (b) Indicate number of participants with missing data for each variable of interest |  |  |  |
| Outcome data | 15* | Report numbers of outcome events or summary measures |  | 6 |  |
| Main results | 16 | (*a*) Give unadjusted estimates and, if applicable, confounder-adjusted estimates and their precision (eg, 95% confidence interval). Make clear which confounders were adjusted for and why they were included |  | 6 |  |
|  |  | (*b*) Report category boundaries when continuous variables were categorized |  |  |  |
|  |  | (*c*) If relevant, consider translating estimates of relative risk into absolute risk for a meaningful time period |  |  |  |
| Other analyses | 17 | Report other analyses done—eg analyses of subgroups and interactions, and sensitivity analyses |  | Suppl. |  |
| Discussion | | |  |  |  |
| Key results | 18 | Summarise key results with reference to study objectives |  | 7, 8 |  |
| Limitations | 19 | Discuss limitations of the study, taking into account sources of potential bias or imprecision. Discuss both direction and magnitude of any potential bias |  | 8 |  |
| Interpretation | 20 | Give a cautious overall interpretation of results considering objectives, limitations, multiplicity of analyses, results from similar studies, and other relevant evidence |  | 7, 8 |  |
| Generalisability | 21 | Discuss the generalisability (external validity) of the study results |  | 7, 8 |  |
| Other information | | |  |  |  |
| Funding | 22 | Give the source of funding and the role of the funders for the present study and, if applicable, for the original study on which the present article is based |  | n.a. |  |

*Give information separately for exposed and unexposed groups.

**Note:** An Explanation and Elaboration article discusses each checklist item and gives methodological background and published examples of transparent reporting. The STROBE checklist is best used in conjunction with this article (freely available on the Web sites of PLoS Medicine at http://www.plosmedicine.org/, Annals of Internal Medicine at http://www.annals.org/, and Epidemiology at http://www.epidem.com/). Information on the STROBE Initiative is available at www.strobe-statement.org.

**References**

1. Jaretzki A, Barohn RJ, Ernstoff RM, et al. Myasthenia gravis: recommendations for clinical research standards. Task Force of the Medical Scientific Advisory Board of the Myasthenia Gravis Foundation of America. *Neurology*. 2000;55(1):16-23. doi:10.1212/wnl.55.1.16

2. Sharshar T, Chevret S, Mazighi M, et al. Validity and reliability of two muscle strength scores commonly used as endpoints in assessing treatment of myasthenia gravis. *J Neurol*. 2000;247(4):286-290. doi:10.1007/s004150050585

3. Burns TM, Conaway MR, Cutter GR, Sanders DB. Less is more, or almost as much: a 15-item quality-of-life instrument for myasthenia gravis. *Muscle Nerve*. 2008;38(2):957-963. doi:10.1002/mus.21053

4. Wolfe GI, Herbelin L, Nations SP, Foster B, Bryan WW, Barohn RJ. Myasthenia gravis activities of daily living profile. *Neurology*. 1999;52(7):1487-1489. doi:10.1212/wnl.52.7.1487

5. Chalder T, Berelowitz G, Pawlikowska T, et al. Development of a fatigue scale. *J Psychosom Res*. 1993;37(2):147-153. doi:10.1016/0022-3999(93)90081-p

6. Zigmond AS, Snaith RP. The hospital anxiety and depression scale. *Acta Psychiatr Scand*. 1983;67(6):361-370. doi:10.1111/j.1600-0447.1983.tb09716.x

7. Bastien CH, Vallières A, Morin CM. Validation of the Insomnia Severity Index as an outcome measure for insomnia research. *Sleep Med*. 2001;2(4):297-307. doi:10.1016/s1389-9457(00)00065-4

8. Morin CM, Belleville G, Bélanger L, Ivers H. The Insomnia Severity Index: psychometric indicators to detect insomnia cases and evaluate treatment response. *Sleep*. 2011;34(5):601-608. doi:10.1093/sleep/34.5.601
